# A Mild Protest

**Published:** 1890-09

**Authors:** 


### A MILD PROTEST.

UNDER the above title, the *Nashville Journal of Medicine and Surgery*, July, 1890, calls us to account in the following language :

Our much-esteemed contemporary the *Buffalo Medical and Surgical Journal*, under the caption of "Twixt Tweedle-dum and Tweedledee," has been pleased to criticise an editorial that appeared in the May issue of the JOURNAL, starting out in his remarks with the mistake that this journal is the organ of the President-elect of the American Medical Association. We would like to disabuse the mind of the distinguished editor of the fact that the JOURNAL is anybody's organ, for we lay claim to a certain amount of independence of any person or any body. This misconstruction of sentiments expressed in the editorial referred to is in reference to our comments upon the matter of consulting with homeopathic or other irregular practitioners. The following is our language quoted by him : "There can be no doubt that certain circumstances *might be looked upon* as mitigating the stringency of this section of the code, as for example the transference of a surgical case by an irregular to a surgeon, and it would seem that at times such a combination of circumstances have been taken advantage of, surgeons meeting and continuing in consultation with irregulars entrusted to the former by the latter." We have italicized certain words in the extract, that show the conditional nature of the meaning intended to be conveyed. We cannot see at all that the above extract meant in any way favoring consulting or consorting with irregulars. Certainly we did not mean to convey that impression, for individually no one is more positively averse to the recognition of homeopathy in any form than ourselves. As for the JOURNAL being the mouth-piece of the President-elect of the American Medical Association, we wish to enter a mild disclaimer.

We hope our confrère will read the position of the editor of this journal aright.

If our good friend will reëxamine the article of which he complains, he will observe that we reproduced it from the Chicago *Medical Standard*, giving the latter credit therefor. We regret that it should seem to do injustice to our highly respected Nashville contemporary. We do not believe "the *Journal* is anybody's organ," though it was, perhaps, natural for the *Standard* to infer from the relationship of the editor of the *Journal* to the president of the American Medical Association, that the former was entitled to speak *ex cathedra*, to a certain extent at all events, in ethical and other constructive expositions of the law of the association.

We approve most heartily of the position taken by the *Journal* upon the point in question, and desired to be merely so understood when we gave the heading "Twixt Tweedle-dum and Tweedle-dee" to the *Standard's* utterance on the subject, adding at the end "Echo answers, Wherein ?"

---

THE importance of proper attention to the selection of underwear is very great in this capricious and continually changing climate. There is no greater fallacy than the popular belief that silk is the proper texture. Science and experience both agree that woolen properly woven furnishes the correct and healthful dressing for the surface of the body, not only keeping it warm but enabling the skin to act properly — in short, it is the ideal substance with which to construct hygienic underwear. The Harderfold Fabric Company, of Troy, N. Y., sends us a catalogue of its underwear, and from personal experience we can affirm there is no better made. We observe that Adam, Meldrum & Anderson, of Buffalo, are agents for the sale of these garments. Every physician should at least examine them.

